# The use of micro-costing in economic analyses of surgical interventions: a systematic review

**DOI:** 10.1186/s13561-020-0260-8

**Published:** 2020-01-29

**Authors:** Shelley Potter, Charlotte Davies, Gareth Davies, Caoimhe Rice, William Hollingworth

**Affiliations:** 1 Bristol Centre for Surgical Research, Population Health Sciences, Bristol Medical School, Room 2.05, Canynge Hall, 39 Whatley Road, Clifton, Bristol BS8 2PS UK; 2 Bristol Breast Care Centre, North Bristol NHS Trust, Southmead Road, Bristol, BS10 5NB UK; 3 Population Health Sciences, Bristol Medical School, University Of Bristol, Southmead Road, Bristol, BS8 2PS UK

**Keywords:** Micro-costing, Economic evaluation, Surgery, Systematic review

## Abstract

**Background:**

Compared with conventional top down costing, micro-costing may provide a more accurate method of resource-use assessment in economic analyses of surgical interventions, but little is known about its current use. The aim of this study was to systematically-review the use of micro-costing in surgery.

**Methods:**

Comprehensive searches identified complete papers, published in English reporting micro-costing of surgical interventions up to and including 22nd June 2018. Studies were critically appraised using a modified version of the Consensus on Health Economic Criteria (CHEC) Checklist. Study demographics and details of resources identified; methods for measuring and valuing identified resources and any cost-drivers identified in each study were summarised.

**Results:**

A total of 85 papers were identified. Included studies were mainly observational comparative studies (*n* = 42, 49.4%) with few conducted in the context of a randomised trial (*n* = 5, 5.9%). The majority of studies were single-centre (*n* = 66, 77.6%) and almost half (*n* = 40, 47.1%) collected data retrospectively. Only half (*n* = 46, 54.1%) self-identified as being ‘micro-costing’ studies. Rationale for the use of micro-costing was most commonly to compare procedures/techniques/processes but over a third were conducted specifically to accurately assess costs and/or identify cost-drivers. The most commonly included resources were personnel costs (*n* = 76, 89.4%); materials/disposables (*n* = 76, 89.4%) and operating-room costs (*n* = 62,72.9%). No single resource was included in all studies. Most studies (*n* = 72, 84.7%) identified key cost-drivers for their interventions.

**Conclusions:**

There is lack of consistency regarding the current use of micro-costing in surgery. Standardising terminology and focusing on identifying and accurately costing key cost-drivers may improve the quality and value of micro-costing in future studies.

**Trial registration:**

PROSPERO registration CRD42018099604.

## Background

Accurate information regarding the cost-effectiveness of existing and novel surgical interventions is vital to inform policy and ensure scarce resources are used to optimal benefit. Central to the meaningful assessment of cost-effectiveness is the accurate evaluation of resource use [1]. This is commonly undertaken using a ‘top-down’ or ‘gross-costing’ approach whereby average costs are used to estimate resource use. For instance, in England, national reference costs based on healthcare resource groups (HRGs) can be used to estimate the average cost per inpatient episode for groups of surgical procedures (e.g. “very major hip procedures for non-trauma”) [2]. While this method is straightforward, it is too crude for many purposes. For example, it is not possible to compare the costs of two different surgical procedures within the same procedure group (e.g. cemented vs hybrid total hip replacements) or evaluate a modification to an existing procedure when the procedure itself is unchanged (e.g. single port vs standard multiple port laparoscopic appendicectomy). Furthermore, gross costing approaches may not be suitable for evaluating novel surgical interventions (e.g. robotic cardiac surgery) which do not fall within existing HRGs. Micro-costing is a method that allows for more precise assessment of the economic costs of a healthcare intervention [3]. Defined as the ‘direct enumeration and costing of every input consumed in the treatment of a particular patient’ [4], micro-costing attempts to measure costs of a service as accurately as possible. The process has three stages; the identification of all resources involved in the provision of care (e.g. human-resources/theatre-time/consumables); accurate measurement of each resource (for example using time-and-motion studies); and valuation of the resources used. Although this approach is time-consuming, it may more accurately reflect the cost of surgical procedures, especially if the procedure is new or includes the use of expensive implants or consumables. It may therefore be the most appropriate method of resource use assessment alongside surgical trials.

The potential value of this methodology has already been identified and a framework has been developed for applying micro-costing methodology to cost evaluations of surgical technologies [5]. This framework focuses specifically on direct costs which reflect the price of resources directly attributable to the procedure rather than indirect costs (e.g. overheads) which have to be estimated using an allocation formula [5]. The framework divides the direct costs into two categories; fixed costs which do not vary according to the level of activity (e.g. personnel costs and medical devices e.g. robotic systems) and variable costs such as reusable instruments and disposables which vary according to the type or number of procedures performed. A recent review of use of micro-costing in bariatric surgery suggests that this framework may be more widely applicable to surgical interventions [6], but further work is required to determine whether it is generalisable to a range of surgical procedures.

Despite the potential benefits of micro-costing, it is time and resource intensive and this may limit its utility in clinical trials. Work is therefore needed to explore whether a more targeted approach may be possible. It is hypothesised that surgical interventions will have ‘cost-drivers’ which create or drive the majority of the cost of the activity. If these are consistent across surgical interventions or can be easily identified, it may be possible to develop a simplified micro-costing framework which could be used in future surgical trials.

The aim of this systematic review was therefore to identify and critically appraise published studies reporting the use of micro-costing in economic analyses of surgical interventions to explore the resources included in each study; the methods used for measuring and valuing resource use and reported cost-drivers of surgical interventions to inform recommendations for the future use of micro-costing in surgical interventions.

## Methods

The protocol for this systematic review was registered on the PROSPERO International prospective register of systematic reviews (reference number CRD42018099604) before data extraction commenced.

### Literature search strategy

The OVID SP versions of MEDLINE, Embase, EconLit, the Cochrane Database and the NHS Economic Evaluation Database (NHS EED) were searched using published micro-costing search strategies [6–8] and appropriate search terms for ‘surgery’ developed in collaboration with an information specialist (Additional file 1). The combined search strategy was tested iteratively to ensure the sensitivity in identifying micro-costing studies known to the authors.

The search was limited to human studies, published in English from database inception up to and including 22nd June 2018. Abstracts and conference reports were excluded due to difficulty assessing incomplete information.

Duplicate records were excluded, and titles and abstracts of remaining citations were screened for eligibility using prespecified inclusion criteria (see below). The reference lists of identified studies and existing reviews were also manually searched to identify any additional relevant papers.

### Selection of papers

Full papers reporting the use of micro-costing as a method of resource-use assessment in economic analyses of surgical interventions were eligible for inclusion.

For the purposes of the review, a surgical intervention was defined as any ‘procedure involving an incision with instruments usually performed in an operating theatre and normally involving anesthesia and/or respiratory assistance’ [9]. Micro-costing was defined as the ‘direct enumeration and costing of every input consumed in the treatment of a particular patient’ [4]. Initial scoping demonstrated that many studies stated having undertaken a micro-costing exercise but did not report any details of the resources included or how these resources had been measured or valued. Such studies were not considered informative so only those considered to represent a ‘sufficiently-detailed micro-costing exercise’ were eligible for inclusion.

The definition of a ‘sufficiently detailed’ micro-costing study was iteratively modified during the pilot abstract screening and data extraction phase which included approximately 100 abstracts and 10 papers respectively. The piloting and iterative modification of the abstract screening and data extraction pro-formas were completed by 2 reviewers (CD/SP) following discussion with experienced health economists (WH/CR). For the purposes of this review, a ‘sufficiently-detailed micro-costing study’ was agreed to be one in which i) the elements of the surgical procedure were sufficiently disaggregated and at the very least two separate elements of the procedure (e.g surgeon costs and consumables) costs presented separately AND ii) the unit cost of at least one element of the procedure (e.g. cost per minute of surgeon time; cost per implant) had been considered. Any studies not meeting both of these criteria were excluded.

Economic analyses using ‘top down’ or gross-costing approaches (e.g. HRGs), economic models, conceptual papers, reviews, commentaries, letters, abstracts, editorials and studies not evaluating surgery were excluded. Studies comparing surgery with a non-surgical comparator were included provided that micro-costing of the surgical intervention had been performed and met the inclusion criteria.

All papers were screened for inclusion by two reviewers (CD and SP/GD) using a standardised two-stage screening proforma (Additional file 2). Phase 1 (abstract screening) focused on identifying original full papers reporting economic analyses of surgical interventions using a micro-costing approach. Papers meeting these inclusion criteria or in which there was uncertainty proceeded to phase 2 (full-text screening). Only papers meeting all the inclusion criteria following full-text screening were included in the review. Uncertainties that remained after full-text review were resolved by discussion with an experienced health economist (CR/WH). Reasons for exclusion were recorded.

### Data extraction

Data were extracted using a standardised REDCap [10] data extraction proforma and included three sections; i) study and surgical procedure characteristics, ii) critical appraisal and iii) details of the micro-costing methodology reported (Additional file 3).

#### Study and surgical procedure characteristics

Study details included year of publication and country of origin; study design (randomised controlled trial or observational study with or without comparison group); prospective or retrospective accrual of data; author-reported type of economic analysis undertaken (e.g. micro-costing; cost analysis, cost-effectiveness analysis; economic analysis; other, irrespective of whether this was considered appropriate); number of participating centres (in the micro-costing study separately); surgical specialty with details of the intervention(s) assessed; whether implants were used (yes or no); type of hospital stay (day-case; inpatient, both or not stated); type of anaesthetic (general, local, both or not stated); details of the study population and stated aims and objectives of the study which were extracted verbatim.

#### Critical appraisal

In the absence of a specific quality assessment tool for micro-costing studies, a modified version of the Consensus on Health Economic Criteria (CHEC) Checklist [11] was used to assess the quality of included studies. The CHEC checklist represents a generic core set of 19 items that can be used to assess the methodological quality of economic evaluations. As this review focuses specifically on the use of micro-costing in economic analyses of surgical interventions, items relating to the appropriateness of the selected time horizon (item 5); the quality of outcome assessment (items 10–12) and the appropriate discounting of future costs and outcomes (item 14) were not considered relevant and were not assessed.

Items 7–9 which assess the degree to which all relevant costs were appropriately identified, measured and valued were the focus of the review and were expanded to include additional details (see below).

#### Reporting of micro-costing methodology

Details of the micro-costing undertaken in each included study were extracted. This included assessing whether the authors reported performing a ‘micro-costing’ study or if alternative terms were used; the purpose of the study (e.g. to compare surgical procedure costs or to compare costing methodologies); methods of resource identification (e.g. patient pathway mapping, interviews with medical staff,) and the scope of the costing exercise (e.g. pre-operative planning; surgical procedure; post-operative hospital stay).

Resources included in each micro-costing study were recorded (yes/no) using the categories proposed in the existing framework [5] expanded based on initial scoping work and expertise within the study team. These included costs relating to personnel; materials/disposables; medical devices; reusable instruments; the operating room; inpatient hospital stay; overhead/administration; drugs/medications; investigations/imaging; complications; follow-up and other costs. For each resource category, specific details of what resources were included (e.g. surgeon, nursing and anaesthetist time in ‘personnel costs’); and details of how these resources were i) measured (e.g. interviews; time and motion studies) and ii) valued (e.g. invoice amounts; hospital human resources departments; provider price catalogues) were extracted verbatim. Finally, we recorded any cost drivers identified by the authors (yes/no) with verbatim details of cost-drivers reported. Most studies did not report results in sufficient detail for us to apply a quantitative definition to identify ‘cost drivers’ or compare their relative importance between studies. Therefore, we are limited to providing a narrative summary of author-identified cost drivers. Similarly, there was insufficient detail to determine whether the reported studies distinguished adequately between static cost drivers, accounting for the highest proportion of total cost at a given point in time, and dynamic cost drivers, making the main contribution to the growth of total costs over time.

Data extraction was performed by one reviewer (CD) with a proportion (10%) checked by a second reviewer (SP/GD). Discrepancies were resolved by discussion with an experienced health economist (CR/WH).

### Analysis

Descriptive statistics were used to summarise study details; compliance with items in the CHEC checklist and details of the micro-costing reported in each study. Simple content analysis [12] was used to categorise verbatim data relating to study aims and objectives; methods used for identifying, measuring and valuing resources and details of any reported cost-drivers. Stata/MP 15 was used for all quantitative analyses.

## Results

### Study selection

**Fig. 1:**
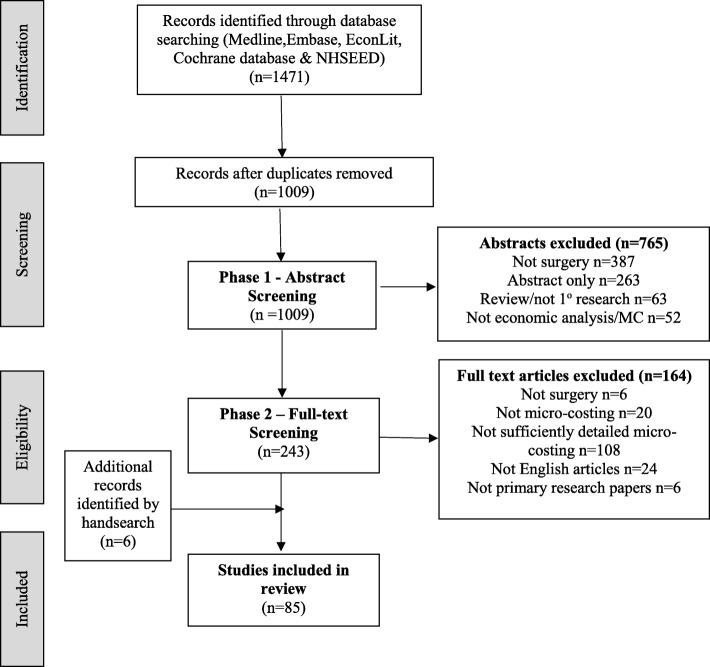
PRISMA diagram for the systematic review

Of the 1009 abstracts identified from the electronic searches, 243 full papers were obtained for further evaluation. Of these, 79 met the inclusion criteria and were retained. A further six papers were identified from the hand-search. A total of 85 papers were therefore included in the review (Fig. 1). Included papers are listed in Additional file 4.

### Study and procedure characteristics

The characteristics of the included studies are summarised in Table 1. Studies were most frequently published between 2016 and 2018 (*n* = 34, 40.0%) and the majority originated from Europe (*n* = 41, 48.2%) or North America (*n* = 21, 24.7%). Included studies were commonly single-centre (*n* = 66, 77.6%), retrospective (*n* = 40, 47.1%), observational studies with (*n* = 46, 54.1%) or without (*n* = 21, 24.7%) a comparison group. Authors’ most frequently described their studies as being ‘cost analyses’ (*n* = 54, 63.5%) and the median number of procedures micro-costed per study was 100 (interquartile range 24–233) although this ranged widely (Table 1).

**Table 1:** Study and surgical procedure characteristics (*n* = 85)

	*n* (%)
Year of publication	
Pre 2000	4 (4.7)
2001–2005	5 (5.9)
2006–2010	11 (12.9)
2011–2015	31 (36.5)
2016–2018	34 (40.0)
Study design	
Within/as part of RCT	5 (5.9)
Observational comparative study	42 (49.4)
Case/control study	4 (4.7)
Case-series	21 (24.7)
Other	13 (15.3)
Data collection	
Prospective	27 (31.8)
Retrospective	40 (47.1)
Combination of prospective and retrospective	4 (4.7)
Not clear/not stated	14 (16.5)
Type of economic analysis reported by study authors	
Cost analysis	54 (63.5)
Micro-costing/activity-based costing	13 (15.3)
Cost-effectiveness analysis	6 (7.1)
Economic analysis	4 (4.7)
Cost Consequence Analysis	3 (3.5)
Cost-utility analysis	1 (1.2)
Other	4 (4.7)
Country of origin	
Europe	41 (48.2)
USA	21 (24.7)
UK	7 (8.2)
Canada	4 (4.7)
Other	6 (7.1)
Multinational	6 (7.1)
Number of participating centres	
Single centre	66 (77.6)
Multicentre	16 (18.8)
Not clear/not stated	3 (3.5)
Number of patients/procedures micro-costed (median, interquartile range, range)	100 (24–233) (6–2130)
Surgical speciality	
Orthopaedics	22 (25.9)
General surgery	14 (16.5)
Plastic surgery	9 (10.6)
Obstetrics and gynaecology	8 (9.4)
ENT	7 (8.2)
Maxillofacial surgery	5 (5.9)
Cardiothoracic surgery	5 (5.9)
Urology	3 (3.5)
Neurosurgery	2 (2.4)
Vascular surgery	1 (1.2)
Other	4 (4.7)
Surgical procedure involving an implant	25 (29.4)
Type of anaesthesia used	
General anaesthesia only	39 (46.4)
Local anaesthesia only	4 (4.8)
Both local and general anaesthesia	5 (6.0)
Not stated/not clear	36 (42.9)
Type of hospital stay	
Day-case procedures	13 (15.3)
Inpatient procedures	43 (50.6)
Both day-case and inpatient procedures	11 (12.9)
Not stated/not clear	18 (21.2)

Orthopaedic (*n* = 22, 25.9%), general (*n* = 14, 16.5%) and plastic surgical (*n* = 9, 10.6%) procedures were micro-costed most frequently but a comprehensive range of surgical specialities were represented and almost a third of studies micro-costed a surgical procedure involving an implant (*n* = 25, 29.4%). Most procedures were performed under general anesthesia (*n* = 39, 46.4%) and required an inpatient hospital stay (*n* = 43, 50.6%) but this information was often not clearly reported (Table 1).

### Critical appraisal

The majority of studies scored highly according to the CHEC checklist (Table 2). Included studies largely had a well-designed research question (*n* = 85, 100%) in a clearly defined population (*n* = 84, 98.8%) and were using an appropriate economic study design to achieve their stated objectives (*n* = 85, 100%). The study perspective was less well-reported (*n* = 49, 57.6%) and only a quarter of studies (*n* = 23, 27.1%) performed any form of sensitivity analysis. Most studies discussed the generalisability of the results to other settings and patient/client groups (*n* = 76, 89.4%). A quarter of studies (*n* = 21, 24.7%) did not report any conflicts of interest; only half (*n* = 43, 50.6%) reported obtaining ethical approval for the project.

**Table 2:** Quality assessment of included studies

	*n* (%)
Is the study population clearly defined?	
Yes	84 (98.8)
No	1 (1.2)
Are the competing alternatives clearly described?	
Yes	63 (74.1)
Not applicable	22 (25.9)
Is a well-designed research question posed in an answerable form?	
Yes	85 (100.0)
Is the economic study design appropriate to the stated objective?	
Yes	85 (100)
Is the actual perspective chosen appropriate?	
Yes	48 (56.5)
No	1 (1.2)
Not stated	36 (42.4)
Sensitivity analysis performed	
No sensitivity analysis performed	61 (71.8)
Deterministic sensitivity analysis	18 (21.2)
Stochastic sensitivity analysis	2 (2.4)
Other stated sensitivity analysis	3 (3.5)
Not stated/not clear	1 (1.2)
Do the conclusions follow from the data reported?	
Yes	85 (100)
Does the study discuss the generalizability of the results to other settings and patient/client groups?	
Yes	76 (89.4)
No	9 (10.6)
Do the authors reports any conflict of interest?	
Yes	7 (8.2)
No	57 (67.1)
Not stated	21 (24.7)
Was ethical approval obtained for the study?	
Yes	43 (50.6)

#### Reporting of micro-costing methodology

Table 3 summarises details of the micro-costing undertaken in the included studies. There was a lack of consistency regarding study terminology with just over half of authors (*n* = 46, 54.1%) describing their methodology as ‘micro-costing’ while a third (*n* = 27/85, 31.8%) used the term (time-driven) activity-based costing (ABC) and a smaller number referred to using a ‘bottom-up’ approach (*n* = 4/85, 4.7%).

**Table 3:** Reporting of micro-costing methodology

	*n* (%)
Identified as authors as being a micro-costing study?	
Yes	46 (54.1)
No	39 (45.9)
If no, authors’ description of their methodology (*n* = 39)	
Activity-based costing or time driven activity-based costing	27 (69.2)
Bottom up approach	4 (10.3)
Direct/detailed cost calculation	3 (7.7)
Unit costs	2 (5.1)
Cost analysis	1 (2.6)
Other/Unclear	2 (5.1)
Stated aim of the microcosting study^a^	
To compare procedures/techniques/processes	43 (50.6)
To determine accurate costs/identify cost drivers	29 (34.1)
To compare costing methodologies (e.g. micro-costing and HRGs)	20 (23.5)
Other stated	2 (2.3)
What aspects of the patient pathway were micro-costed	
Pre-operative planning/investigations	48 (56.5)
Surgical procedure	85 (100.0)
Hospital stay	72 (84.7)
Complications of surgery	29 (34.1)
Follow up	29 (34.1)
Rehabilitation (physiotherapy/occupational therapy)	6 (7.1)
Other	1 (1.2)
Separate reporting of input utilisation and unit cost data	
Yes	50 (58.8)
No	35 (41.2)
Did the authors report both direct and indirect costs	
Direct costs only	24 (28.2)
Both direct and indirect costs	29 (34.1)
Not stated	32 (37.6)
Methods by which resources were identified^b^	
Patient pathway mapping	32 (37.6)
Interviews with surgeons/patients	26 (30.5)
Accounting/finance department	17 (20)
Hospital information systems/administrative databases	37 (43.5)
Direct observation	20 (23.5)
Review of patient notes/charts	16 (18.8)
Review of operating logs/books	5 (5.9)
Standardised reporting template	2 (2.3)
Manufacturer	2 (2.3)
Case report forms	1 (1.2)
Other	11 (12.9)
Resources identified and reported^c^	
Personnel costs	76 (89.4)
Materials/disposables	76 (89.4)
Medical device costs	34 (40.0)
Re-usable instrument costs	16 (18.8)
Operating room costs (separate from admission costs)	62 (72.9)
Inpatient hospital stay costs	52 (61.1)
Overhead/administration costs	46 (54.1)
Medicinal/Drug costs	63 (74.1)
Imaging/investigation/blood tests costs	39 (45.8)
Complications	5 (5.8)
Outpatient/Follow up	9 (10.5)
Perioperative care (pre-operative care/recovery)	4 (4.7)
Other costs	10 (11.7)
Did the authors identify cost drivers	
Yes	72 (84.7)
No	13 (15.3)

*HRG* healthcare resource groups

^a^Some studies had more than one stated micro-costing aim

^b^Most studies report more than one method

^c^Most studies report more than one resource

Micro-costing was most commonly performed to compare procedures, techniques or processes (*n* = 43, 50.6%) but over a third of studies (*n* = 29, 34.1%) were specifically undertaken to identify cost-drivers for surgical procedures and a quarter (*n* = 20, 23.5%) were performed to compare costing methodologies; most commonly gross or ‘top-down’ methods, such as HRGs or insurance reimbursement with micro-costing or ‘bottom-up’ approaches. In addition to micro-costing the actual surgical procedure, most studies (*n* = 72, 84.7%) also micro-costed the patients’ hospital stay. Pre-operative investigations required prior to surgery were included in over half the studies (*n* = 48, 56.5%) and a third micro-costed surgical complications (*n* = 29, 34.1%) and/or follow-up required after discharge (*n* = 29, 34.1%). Most studies reported including either direct costs only (*n* = 24, 28.2%) or both direct and indirect costs (*n* = 29, 34.1%) but over a third (*n* = 32, 37.6%) did not state what types of costs had been considered.

Although most studies included personnel costs (*n* = 76, 89.4%); materials and disposables (*n* = 76, 89.4%); operating room costs (*n* = 62, 72.9%) and/or the costs of any drugs or medications (*n* = 63, 74.1%) in their micro-costing, there was no single type of resource included in all studies and categorisation of resources into pre-defined categories was often difficult due to different studies aggregating and reporting resources in different ways. There was also a lack of consistency in the ways in which relevant resources were identified, measured and valued. Hospital information systems or administrative databases were used in over 40% (*n* = 37, 43.5%) of studies with approximately a third (*n* = 32, 37.6%) using patient pathway mapping to identify resource-use. Interviews with surgeons and/or patients (*n* = 26, 30.5%) and direct observation (*n* = 20, 23.5%) were less commonly used (Table 3).

#### Identification of cost-drivers

The majority of included studies (*n* = 72, 84.7%) reported having identified cost-drivers but these differed according to whether the authors had micro-costed the surgical procedure alone or the whole episode of care (surgical procedure including the inpatient stay +/− preoperative investigations +/− follow-up) (Table 4). For studies just considering the surgical procedure, the main cost-drivers were identified as the costs of the operating theatre (*n* = 17, 28.3%); theatre personnel (*n* = 15, 25.0%); operative equipment (*n* = 9, 15.0%), implants (*n* = 7, 11.7%) and theatre consumables (*n* = 6, 10.0%). For studies micro-costing the full episode of care, the main cost-drivers identified were the inpatient stay (*n* = 25, 35.7%) and personnel/labour costs (*n* = 14, 20%). Use of intensive care (*n* = 7, 10%); consumables (*n* = 6, 8.6%) and overheads (*n* = 6, 8.6%) were also identified as potential cost-drivers in these studies (Table 4).

**Table 4:** Main cost drivers identified by included studies^a^ (*n* = 72)

Main Cost-drivers	Cost drivers within the surgical procedure alone (n, %)	Cost drivers within the full episode of care (*n,* %)
Operating room/theatre cost (surgery cost/operating room time)	17 (28.3)	0 (0.0)
Personnel/labour costs	15 (25.0)	14 (20.0)
Operative equipment (disposable and non-disposable, including robots)	9 (15.0)	0 (0.0)
Implant/device used in procedure	7 (11.7)	0 (0.0)
Consumables	6 (10.0)	6 (8.6)
Medications (including blood)	4 (6.6)	5 (7.0)
Inpatient stay	NA	25 (35.7)
Intensive care	NA	7 (10.0)
Physiotherapy and rehabilitation	NA	3 (4.3)
Overheads	0 (0.0)	6 (8.6)
Complications	2 (3.3)	0
Pre-operative visits	NA	1 (1.4)
Medical aids	NA	1 (1.4)
Imaging and diagnostic tests	NA	1 (1.4)
Other	0 (0.0)	1 (1.4)

^a^Main cost drivers reported by authors. Other cost driver were identified but these were stated as the ‘main’ or most important ones

## Discussion

Micro-costing may offer a more accurate method for costing in economic analyses of surgical interventions, but this review suggests that current use of the methodology is inconsistent and, in many instances, lacking in methodological rigor. There is a lack of consistency regarding terminology used; the types of resources included and the methods by which these are measured and valued. Almost half of studies used retrospectively collected data (e.g. from hospital administrative or information systems). Although routinely available data sources may be accurate, especially in modern ‘fee-for-service’ settings, other reported methods for resource use identification such as case-note review or operating room logs may be less robust [13–15], potentially compromising the validity of the micro-costing approach. Over three quarters of all studies were undertaken at a single centre limiting the generalizability of the results. Almost half of the included studies did not report resource utilization and unit cost data separately. Instead summary costs were presented with limited disaggregation of individual costs or transparency regarding precisely which resources had been included making meaningful interpretation difficult. Some good examples of micro-costing surgical interventions, however, were identified [16, 17] and have subsequently been published [18].

There was significant variation in which aspects of the patient pathway researchers elected to micro-cost. Most studies costed both the surgical procedure and the associated inpatient stay, but others also included pre-operative planning/investigations, complications and follow-up required following discharge. To an extent, this variation may appropriately reflect the diversity in the aims of micro-costing studies. For economic evaluations comparing two different surgical interventions researchers may legitimately focus on the incremental cost differences between the two procedures and exclude costs that are common to both procedures (e.g. pre-operative visits). Furthermore, for economic evaluation, methodological guidelines emphasise that the level of resource use detail required is lower for items with a small relative impact on total costs (e.g. volatile anaesthetic agents) [19]. In contrast, for studies aiming to compare the actual costs of surgery to “top down” costs (e.g. hospital reimbursement), it is important to include all elements contributing to cost. The level of detail may also be constrained by study setting and in particular the granularity of the unit costs available for valuing resources. Studies in settings with itemised fee for service hospital reimbursement have greater scope to cost resource use in much finer detail. Approximately one in four studies compared a measured cost of performing a procedure, as determined by micro-costing, with procedure reimbursement rates, which were often considered to be insufficient. Many of these studies were undertaken by clinicians. The scope of micro-costing (e.g. procedure based or full admission) and the economic perspective (hospital or wider health service) in such studies should be rigorously appraised to ensure it is not biased by clinicians’ financial interests. Cost-drivers were identified and reported in most studies, but these differed according to which aspects of the patient pathway were included. There is a need for a standardized definition of a cost driver in the literature so that authors are consistent with this terminology and how it is measured and reported. Although, it was clear from the reported studies in this review that the cost drivers were the resources that involved the highest proportion of the total costs of the surgical pathway and/or surgical procedure, this proportion may in some studies have been below 50% of total cost and/or may have subsumed several cost drivers instead of only one cost driver per pathway or procedure.

This review has demonstrated the need for specific guidance for researchers undertaking micro-costing. Work to develop a checklist for the conduct, reporting and appraisal of micro-costing studies in healthcare is ongoing [20]. This checklist may improve the quality of future studies but is not specific to surgery which presents unique challenges. A framework for costing surgical technologies [5] provides formulae for estimating the fixed (device and personnel) and variable (re-usable equipment and disposables) costs of surgery. However, the framework only considers the costs of the operative procedure which limits its applicability for trials of surgical procedures likely to have resource-use implications beyond the operating theatre such as differential length of inpatient stay; use of intensive care or need for follow-up investigations. The framework also excludes indirect costs (overheads) of surgery which are necessary for micro-costing analyses wishing to compare surgical care with non-surgical care or compare the cost of surgery with procedure reimbursement values. Further work is therefore needed to develop more simplified recommendations for the use of micro-costing in surgery generally and specifically for efficient and effective use of the methodology in surgical trials. Recommendations based on the findings of this review are summarised in Table 5.

**Table 5:** Recommendations for the efficient use of micro-costing as a method of resource-use assessment in surgery

Key Recommendations	
1. Consistently use the term ‘micro-costing’ when describing the methodology and include ‘micro-costing’ in the abstract and as a keyword to facilitate future identification of studies	
2. Identify the potential key cost-drivers (see Table 4) for the surgical intervention based on the research question. Patient pathway mapping with experts (e.g. surgeons and other healthcare professionals) may help identify key resources.	
3. For comparative cost analyses, more accurate albeit time consuming methods (e.g observation) are warranted for resources that differ between comparator procedures, whereas cruder methods (e.g expert opinion) may be sufficient for inexpensive resources with similar use between procedures.	
4. Ensure transparent reporting of micro-costing studies with sufficient disaggregation of elements of the procedure/pathway and reporting of unit costs (in supplementary material if necessary)	
5. Consider applying focused cost-driver micro-costing at multiple centres to improve generalisability of the results	

This is, to our knowledge, the first study to systematically identify and critically-appraise the use of micro-costing as a method of resource-use assessment in surgery. We used published search strategies for micro-costing [6, 7], however due to the lack of standardisation in micro-costing terminology and excluding non-English language papers, our search may have overlooked other relevant studies. The review was restricted to studies meeting our prespecified definition of being a ‘sufficiently-detailed micro-costing exercise’. This led to the exclusion of a large number of studies at the full-text screening stage. Inclusion of these studies would not have been informative, but it is important to note that costing studies in surgery more generally may be methodologically less robust than those included in this review. The majority of included studies scored highly on the CHEC checklist despite an objective lack of methodological rigor. This highlights the need for specific recommendations for the design, conduct and critical appraisal of micro-costing studies to improve the quality and value of this work.

Micro-costing produces an accurate assessment of resource-use but the methodology is time consuming and resource intensive and universal application alongside surgical trials would not be practical [3, 21]. A more targeted or hybrid micro-costing approach, however, may have value [22]. Almost all studies included in the review identified one or more ‘cost-drivers’ that represented the greatest proportion of the costs for their procedures. These differed according to the aspects of the patient pathway considered as for example, operating room costs were the main cost-driver in studies just costing the surgical procedure but were less important in studies costing the full episode of care. Cost-drivers may also be influenced by other factors such as the specific research question and the intervention under study. For example, a study comparing the cost of two surgical procedures identical except for the implant used might reasonably focus on implant cost and detailed observations of surgeon time spent inserting the implant. Focusing attention on identifying and accurately costing these key elements of care while applying less detailed methods (e.g. record review or expert opinion) for other components may allow the benefits of micro-costing to be realized in an efficient way. Process mapping with experts including surgeons, nurses, other allied healthcare professionals and if appropriate, patients themselves may be an effective way to identify key cost elements. More targeted, efficient micro-costing of key cost-drivers may also allow this methodology to be applied over a wider number of centres in a trial setting improving both the accuracy of the costing data obtained and the generalizability of the results [23]. Further work, however, is needed to determine if this targeted approach would be feasible alongside a surgical trial.

As health systems and technologies become more advanced, routinely-available data may have increasing utility for resource-use assessment and may make future large-scale micro-costing studies possible. Healthcare provider cost-accounting systems that capture details on all resources used in an episode of care combined with electronic theatre systems that could provide proxy time and motion data may represent a time and cost-efficient method for detailed resource-use assessment. Such systems and methodology, however, would need to be consistent across centres if the costs generated were to be comparable. This may be challenging, especially across different healthcare settings and geographical locations.

Micro-costing has the potential to improve the accuracy of economic analyses of surgical interventions by providing more accurate assessments of resource-use but the overall quality of existing studies is poor. There is a need to improve the consistency and efficiency of micro-costing in surgery if the potential value of the methodology is to be realised. This review highlights a number of ways in which this could be achieved. Using standardised terminology for micro-costing studies; focusing on identifying and accurately costing cost-drivers relevant to the specific research question and transparently reporting disaggregated costs for each included resource may represent a simple strategy for improving the design and delivery of future studies.

## Conclusions

Micro-costing may provide a more accurate method of resource-use assessment in economic analyses of surgical interventions and could improve the value of economic evaluations conducted alongside surgical trials, but this systematic review suggests that current use of micro-costing in surgery is inconsistent and lacking in methodological rigor. Using standardised terminology for micro-costing studies; focusing on identifying and accurately costing cost-drivers relevant to the specific research question and transparently reporting disaggregated costs for each included resource may represent a simple strategy for the optimal design and delivery of future studies.

## Supplementary information

**Additional file 1.** Search strategy for Ovid Medline (1946 to present) (search 1 to 21 ref Doble et al 2017).

**Additional file 2.** Screening log (Phase 1 and 2).

**Additional file 3.** Data Extraction Form.

**Additional file 4 **Papers included in the systematic review (*n* = 85).

## Data Availability

Data included in this paper will be made available on reasonable request from the corresponding author when all analyses have been completed.

## Abbreviations

ABC: Activity-based costing
CHEC: Consensus on Health Economic Criteria
HRGs: Healthcare resource groups
NHS EED: NHS Economic Evaluation Database
