# Supplementary material for: The use of micro-costing in economic analyses of surgical interventions: a systematic review
Source: Health Econ Rev. 2020 Jan 29;10:3. doi: 10.1186/s13561-020-0260-8 (PMC6990532; doi:10.1186/s13561-020-0260-8)
Supplement: Supplementary file 1 — Additional file 1. Search strategy for Ovid Medline (1946 to present) (search 1 to 21 ref Doble et al 2017). [file 13561_2020_260_MOESM1_ESM.docx]

**Additional file 1: Search strategy for Ovid Medline (1946 to present) (search 1 to 21 ref Doble et al 2017)**

| 1. exp "costs and cost analysis"/ or exp health care costs/ or exp health expenditures/ or exp hospital costs/ 2. (“cost$” or “cost$ study”).ab,hw,kf,kw,ot,sh,ti,tw. |
| --- |
| 1. Bottom-up.ab,hw,kf,kw,ot,sh,ti,tw. 2. (microcost$ or micro-cost$).ab,hw,kf,kw,ot,sh,ti,tw. 3. (bottom-up adj5 (cost$ or accounting)).ab,hw,kf,kw,ot,sh,ti,tw. 4. (activity-based adj5 (cost$ or accounting)).ab,hw,kf,kw,ot,sh,ti,tw. 5. time study.ab,hw,kf,kw,ot,sh,ti,tw. 6. ((time-and-motion or time-motion) adj (study or studies or method* or analys*)).ab,hw,kf,kw,ot,sh,ti,tw. 7. 3 or 4 or 5 or 6 or 7 or 8 8. (1 or 2) and 9 9. exp Specialties, Surgical/ 10. exp Surgical Procedures, Operative/ 11. exp "prostheses and implants"/ 12. exp General Surgery/ 13. (surgery or surgical or surgeon*).ab,hw,kf,kw,ot,sh,ti,tw 14. (operation or operative or post-operative or postoperative).ab,hw,kf,kw,ot,sh,ti,tw. 15. surgery/ 16. 11 or 12 or 13 or 14 or 15 or 16 or 17 17. 10 and 18 18. Animals/ not (Animals/ and Humans/) 19. 19 not 20 |
