# Supplementary material for: The use of micro-costing in economic analyses of surgical interventions: a systematic review
Source: Health Econ Rev. 2020 Jan 29;10:3. doi: 10.1186/s13561-020-0260-8 (PMC6990532; doi:10.1186/s13561-020-0260-8)
Supplement: Supplementary file 2 — Additional file 2. Screening log (Phase 1 and 2). [file 13561_2020_260_MOESM2_ESM.pdf]

# Screening log (Phase 1 and 2)

Record ID ____________________

## Screening Items Phase 1 (abstract)

Reviewer name

◯ Charlotte Davies
◯ Gareth Davies
◯ Shelley Potter

Endnote ID (author surname, year, #number) ____________________

Original research article ◯ Yes ◯ No ◯ Unclear

Full length article ◯ Yes ◯ No

If not full text, what type of publication?
◯ Abstract only
◯ Systematic or narrative review
◯ Letter
◯ Editorial
◯ Study protocol
◯ Other

Surgical procedure(s)/intervention ◯ Yes ◯ No ◯ Not clear

Economic evaluation or cost analysis ◯ Yes ◯ No ◯ Not clear

Applied micro-costing ◯ Yes ◯ No ◯ Not clear

All Phase 1 screening criteria met for full text review ◯ Yes ◯ No ◯ Not clear

If Phase 1 not clear - full text review needed ◯ Yes ◯ No

## Screening Items Phase 2 (full text)

Able to access full text article ? ◯ Yes ◯ No ◯ Need inter lib loan

Have the full text article? ◯ Yes ◯ No

Is study related to surgery? ◯ Yes ◯ No

Is study related to micro-costing? (not micro-costing if e.g a modeling study, top down study) ◯ Yes ◯ No

| | |
|---|---|
| For the SURGICAL PROCEDURE, does the study represent a sufficiently detailed micro-costing exercise? (i.e is at least one component of the procedure microcosted?) Defined by definitions A and B below)<br><br>A. Are the elements of a surgical procedure sufficiently disaggregated and costs of each item presented separately (i.e. at the very least TWO separate elements of PROCEDURE costs presented separately e.g. personnel costs and consumable costs). (look in results for tables)<br><br>B. Have the authors indicated that they have assessed/measured/calculated the unit cost of AT LEAST ONE ELEMENT of procedure | ◯ Yes ◯ No ◯ Not sure |
| Include study in final data extraction? | ◯ Yes ◯ No ◯ Unsure |
| Reason for uncertainty | __________________________________ |
| Give reason for exclusion | __________________________________ |
