# Supplementary material for: The use of micro-costing in economic analyses of surgical interventions: a systematic review
Source: Health Econ Rev. 2020 Jan 29;10:3. doi: 10.1186/s13561-020-0260-8 (PMC6990532; doi:10.1186/s13561-020-0260-8)
Supplement: Supplementary file 3 — Additional file 3. Data Extraction Form. [file 13561_2020_260_MOESM3_ESM.pdf]

Data Extraction Form

Record ID ______________________________

Record ID ______________________________

Endnote ID (author surname, year, #number)
(e.g Smith,2013#3655) ______________________________

Reviewer Name

◯ Charlotte Davies
◯ Gareth Davies
◯ Shelley Potter

Date Extracted ______________________________

Part 1 Study Demographics

Year of publication ______________________________

Study type and design

◯ Randomised controlled trial
◯ Observational comparative study
◯ Case/control study
◯ Case-series (observational, no comparison group)
◯ Other
◯ Not clear

If other, please state study design

____

Data collection

◯ Prospective
◯ Retrospective
◯ Combination of prospective and retrospective
◯ Unclear/Not stated

Type of economic analysis (as described in study by authors)

◯ Cost analysis
◯ Economic analysis
◯ Cost-effectiveness analysis
◯ Cost utility analysis
◯ Cost benefit analysis
◯ Cost minimization analysis
◯ Cost comparison analysis
◯ Cost outcome description
◯ Cost of illness study
◯ Other

If other, please state

____

Country of Origin

◯ UK
◯ Europe
◯ America
◯ Canada
◯ International
◯ Other

If other, please state

____

Number of participating centres (involved In the study as a whole)

◯ Single centre
◯ Multi centre
◯ Not clear, not stated
◯ Other

If other, please state

____

Number of participating centres (in the micro-costing study, if separate to whole study)

◯ Single centre
◯ Multi centre
◯ Not stated/not clear
◯ Other
◯ Not applicable

If other, please state

____

Area

◯ General surgery
◯ Orthopaedics
◯ ENT
◯ Neurosurgery
◯ Paediatric surgery
◯ Other

If other, please state

____

Study intervention: State intervention(s) __________________________________

Study intervention: surgical implant used (e.g mesh) ◯ Yes ◯ No

If yes, please state type of implant

____

Study intervention: type of hospital stay

◯ Daycase
◯ In-patient stay
◯ Both inpatient and daycase procedures
◯ Not stated/not clear

Study intervention: anesthetic used

◯ General anesthetic (GA)
◯ Local anesthetic (LA)
◯ Both GA and LA cases
◯ Not stated/Not clear

Study Population (e.g patients undergoing thyroidectomies (full and partial) performed in study hospital) ____

Sample size : of the whole study __________________________________

Sample size: number of cases or procedures micro-costed __________________________________

Stated aims and objectives of study ____

Part 2 Critical Appraisal

Is the study population clearly defined? ◯ Yes ◯ No ◯ Unsure

Are the competing alternatives clearly described? ◯ Yes ◯ No ◯ Unsure ◯ Not applicable

Is a well-designed research question posed in an answerable form ? ◯ Yes ◯ No ◯ Unsure

Is the economic study design appropriate to the stated objective ? ◯ Yes ◯ No ◯ Unsure

Is the actual perspective chosen appropriate ? ◯ Yes ◯ No ◯ Unsure ◯ Not stated

Sensitivity analysis performed

- ◯ Stochastic (probabilistic) sensitivity analysis
- ◯ Deterministic sensitivity analysis
- ◯ No sensitivity analysis performed
- ◯ Other stated sensitivity analysis
- ◯ Unsure
- ◯ Not applicable

Please give details

____

Do the conclusions follow from the data reported? ◯ Yes ◯ No ◯ Unsure

Does the study discuss the generalizability of the results to other settings and patient/client groups? ◯ Yes ◯ No ◯ Unsure

Do the authors reports any conflict of interest? ◯ Yes ◯ No ◯ Not stated

If yes, what is the stated COI? __________________________________

Are ethical issues discussed appropriately ?

- ◯ Yes
- ◯ No
- ◯ Unsure
- ◯ Ethics approval (or IRB) not mentioned

Part 3 Reporting of micro-costing methodology

Identified by authors as being a micro-costing study ◯ Yes ◯ No

If No, please state authors description of study if not specifically described as a micro-costing study __________________________________

Extent of micro-costing
- ◯ Whole study micro-costed
- ◯ Parts of procedure/pathway micro-costed

Which elements of the study were micro-costed __________________________________

What of aspects of study were NOT micro-costed and what alternative costing methods were used used? __________________________________

Separate reporting of input utilization quantity and unit cost data ◯ Yes ◯ No ◯ Not clear

Did the author report both direct and indirect costs?
- ◯ Direct costs only
- ◯ Both direct and indirect costs
- ◯ Not stated

What cost components were stated as being included in 'direct costs'

____

What cost components were stated as being included in 'indirect costs'

____

Methods by which resources were identified

- ☐ Patient pathway mapping
- ☐ Interviews with surgeon(s) or other medical staff
- ☐ Accounting department
- ☐ Database(s)
- ☐ Direct observation/observation fieldwork
- ☐ Other
- ☐ Review of patient notes or charts
- ☐ Review of operating logs/books
- ☐ Not stated

If other, please state

____

Please provide details

____

Elements of the patient pathway considered

- ☐ Pre-operative planning/investigations
- ☐ Surgical procedure (including anaesthetic)
- ☐ Hospital stay
- ☐ Complications of surgery (if any)
- ☐ Follow up
- ☐ Any rehabilitation (e.g occupational or physiotherapy) required
- ☐ Other
- ☐ Not stated

If other, please state

____

If follow up period included, please state duration and details of follow up

If rehabilitation included, please state duration and details reported

Resources identified (labeled a to j below) as being involved in the provision of care (cost components)

a) Personnel costs reported (e.g surgeon, nurse, anesthesiologist time)

◯ Yes ◯ No

a) Personnel costs reported, please indicate for e.g if surgeon, nurse or anesthesiologist time

☐ Surgeon
☐ Nurse
☐ Anesthesiologist
☐ Operating department practitioner
☐ Other
☐ Not stated

a) If other, please state

____

Please provide details

a) Personnel costs : methods of measurement / method of quantity data collection

☐ time-motion study (direct observation)
☐ casenote/patient record review
☐ cost accounting database
☐ Interviews with surgeons/nurses/other staff
☐ survey of clinicians
☐ expert opinion
☐ other
☐ Not stated

a) If other, please state

____

Please provide verbatim details of methods for MEASURING personnel costs reported in paper

a) Personnel costs : methods used for valuation/ Method of unit cost data collection (consider assessing methods used in paper as a whole)

☐ invoice amount
☐ hospital/clinic/provider price catalogue
☐ national/regional/provincial/hospital/ insurer fee schedule
☐ human resources/payroll record
☐ other
☐ not stated

a) If other, please state

____

Please provide verbatim details of methods for VALUING personnel costs reported in paper

a) Personnel costs : actual stated costs

____

b) Materials/ Disposable instrument/consumables costs reported (e.g needles, disposable staplers)

◯ Yes ◯ No

b) Specify Materials/ Disposable instrument/consumables costs reported (e.g needles, disposable staplers)

b) Materials/Disposable instrument/consumables : methods of measurement / method of quantity data collection

☐ time-motion study/direct observation in theatre
☐ cost accounting database
☐ provider/staff interview
☐ case note/patient record review
☐ survey of clinicians
☐ expert opinion
☐ other
☐ not stated

b) If other, please state

____

Please provide verbatim details of methods for MEASURING materials/instruments/disposables reported in paper

b) Materials/Disposable instrument/consumables: methods used for valuation/ Method of unit cost data collection (consider assessing methods used in paper as a whole)

☐ invoice amount
☐ hospital/clinic/provider/government price catalogue
☐ national/regional/provincial/hospital/insurer fee schedule
☐ Online databases/electronic resources (not institution specific)
☐ other
☐ not stated
☐ Government contracts

b) If other, please state

____

Please provide verbatim details of methods for VALUING materials/instruments/disposables reported in paper

b) Actual stated costs of Materials/Disposable instrument/consumables

____

c) Medical device costs reported (e.g endoscopy column, laparoscopic tower, also includes equipment repair costs)

◯ Yes ◯ No

c) Specify the medical devices reported (e.g endoscopy column, laparoscopic tower, also includes equipment repair costs)

c) Medical device costs : methods of measurement / method of quantity data collection

☐ time-motion study/direct observation in theatre
☐ cost accounting database
☐ provider/staff interview
☐ case note/patient record review
☐ survey of clinicians
☐ expert opinion
☐ other
☐ not stated

c) If other, please specify

____

Please provide verbatim details of methods for MEASURING medical device costs reported in paper

c) Medical device costs: methods used for valuation/ Method of unit cost data collection (consider assessing methods used in paper as a whole)

☐ invoice amount
☐ hospital/clinic/provider/government price catalogue
☐ national/regional/provincial/hospital/ insurer fee schedule
☐ Online databases/electronic resources (not institution specific)
☐ other
☐ not stated

c) If other, please state

____

Please provide verbatim details of methods for VALUING medical device costs reported in paper

c) Actual stated costs of medical devices (e.g endoscopy column, laparoscopic tower, also includes equipment repair costs)

____

d) Re-usable instrument costs reported (e.g bowel graspers, surgical scissors)

◯ Yes ◯ No

Details of re-usable instruments included

d) Re-usable instrument costs : methods of measurement / method of quantity data collection

☐ time-motion study/direct observation in theatre
☐ cost accounting database
☐ provider/staff interview
☐ case note/patient record review
☐ survey of clinicians
☐ expert opinion
☐ other
☐ not stated

d) If other , please state

____

Please provide verbatim details of methods for MEASURING re-usable equipment costs reported in paper

d) Re-useable instrument costs: methods used for valuation/ Method of unit cost data collection (consider assessing methods used in paper as a whole)

☐ invoice amount
☐ hospital/clinic/provider/government price catalogue
☐ national/regional/provincial/hospital/ insurer fee schedule
☐ Online database/electronic record (not institution specific)
☐ other
☐ not stated

d) If other, please state

____

Please provide verbatim details of methods for VALUING re-usable equipment costs reported in paper

d) Actual stated costs of re-usable instrument reported (e.g bowel graspers, surgical scissors)

____

e) Operating room costs reported (separate from hospital admission costs)

◯ Yes ◯ No

e) Operating room costs : methods of measurement / method of quantity data collection

- ☐ time-motion study/direct observation in theatre
- ☐ cost accounting database
- ☐ provider/staff interview
- ☐ case note/patient record review
- ☐ survey of clinicians
- ☐ expert opinion
- ☐ other
- ☐ not stated

e) If other, please state

____

Please provide verbatim details of methods for MEASURING operating room costs reported in paper

e) Operating room costs: methods used for valuation/ Method of unit cost data collection (consider assessing methods used in paper as a whole)

- ☐ invoice amount
- ☐ hospital/clinic/provider/government price catalogue
- ☐ national/regional/provincial/hospital/ insurer fee schedule
- ☐ Online resources/databases (not institution specific)
- ☐ other
- ☐ not stated

e) If other, please state

____

Please provide verbatim details of methods for VALUING operating room costs reported in paper

Details of how operating room is costed

e) Actual stated costs of operating room reported (separate from hospital admission costs)

____

f) Inpatient hospital stay costs reported

◯ Yes ◯ No

f) Inpatient hospital stay costs : methods of measurement / method of quantity data collection

☐ time-motion study (direct observation)
☐ cost accounting database
☐ provider/staff interview
☐ case note/patient record review
☐ survey of clinicians
☐ expert opinion
☐ other
☐ not stated

f) If other, please state

____

Please provide verbatim details of methods for MEASURING inpatient hospital stay costs reported in paper

f) Inpatient hospital stay costs: methods used for valuation/ Method of unit cost data collection (consider assessing methods used in paper as a whole)

☐ invoice amount
☐ hospital/clinic/provider/government price catalogue
☐ national/regional/provincial/hospital/ insurer fee schedule
☐ human resources/payroll record
☐ other
☐ not stated

f) If other, please state

____

Please provide verbatim details of methods for VALUING inpatient hospital stay costs reported in paper

f) Actual stated costs of Inpatient hospital stay

____

g) Overhead and/or Administration costs reported

◯ Yes ◯ No

g) Overhead and/or administration costs : methods of measurement / method of quantity data collection

- ☐ time-motion study
- ☐ cost accounting database
- ☐ provider/staff interview
- ☐ case note/patient record review
- ☐ survey of clinicians
- ☐ expert opinion
- ☐ other
- ☐ not stated

g) If other, please state

____

g) Overhead and/or Administration costs: methods used for valuation/ Method of unit cost data collection (consider assessing methods used in paper as a whole)

- ☐ invoice amount
- ☐ hospital/clinic/provider/government price catalogue
- ☐ national/regional/provincial/hospital/ insurer fee schedule
- ☐ Online/electronic database (not institution specific)
- ☐ other
- ☐ not stated

g) If other, please state

____

Details of how overheads/indirect costs calculated

g) Actual stated costs of overheads and/or Administration

____

h) Medicinal costs (e.g anesthetics) reported

◯ Yes ◯ No

Details of medicinal costs included

____

h) Medicinal costs : methods of measurement / method of quantity data collection

☐ time-motion study (direct observation)
☐ casenote/patient record review
☐ cost accounting database
☐ provider/staff interview
☐ survey of clinicians
☐ expert opinion
☐ other
☐ not stated

h) If other, please state

____

Please provide verbatim details of methods for MEASURING medicinal costs reported in paper

h) Medicinal costs: methods used for valuation/ Method of unit cost data collection (consider assessing methods used in paper as a whole)

☐ invoice amount
☐ hospital/clinic/provider/government price catalogue
☐ national/regional/provincial/hospital/ insurer fee schedule
☐ Electronic/online databases/resources (not institution specific) e.g BNF
☐ other
☐ not stated

h) If other, please state

____

Please provide verbatim details of methods for VALUING medicinal costs reported in paper

h) Actual stated medicinal costs (e.g anesthetics)

____

i) Imaging/Investigations/Blood tests costs

◯ Yes ◯ No

i) Imaging/investigations/Blood Test costs etc : methods of measurement / method of quantity data collection

☐ time-motion study (direct observation)
☐ casenote/patient record review
☐ cost accounting database
☐ provider/staff interview
☐ survey of clinicians
☐ expert opinion
☐ other
☐ not stated

i) If other, please state

____

Please provide verbatim details of methods for MEASURING imaging/investigation/blood test costs reported in paper

i) Imaging/investigations/Blood tests costs etc: methods used for valuation/ Method of unit cost data collection (consider assessing methods used in paper as a whole)

☐ invoice amount
☐ hospital/clinic/provider/government price catalogue
☐ national/regional/provincial/hospital/ insurer fee schedule
☐ Online/electronic database/resources (not institution specific)
☐ other
☐ not stated

i) If other, please state

____

Please provide verbatim details of methods for VALUING imaging/investigation/blood test costs reported in paper

i) Actual stated Imaging/Investigations/Blood tests costs

____

j) Any other resources or costs identified as being involved in the provision of care ?, that do not fall under the following categories:
a) Personnel costs
b) Materials/Disposable instrum/Consumables
c) Medical device costs
d) Re-useable instruments costs
e) Operating room costs
f) Inpatient hospital stay costs
g) Overhead/Admin costs
h) Medicinal costs
i) Imaging/Investigations/Blood tests

◯ Yes ◯ No

j) Please describe this other resource or cost

j) Method of measurement used/ method of quantity data collection ?

☐ time-motion study
☐ casenote/patient record review
☐ cost accounting database
☐ provider/staff interview
☐ survey of clinicians
☐ expert opinion
☐ other
☐ not stated

j) If other, please state

____

Please provide verbatim details of methods for MEASURING other stated resource costs reported in paper

j) Methods used for valuation /Method of unit cost data collection (consider assessing methods used in paper as a whole)

☐ invoice amount
☐ hospital/clinic/provider price catalogue
☐ national/regional/provincial/hospital/ insurer fee schedule
☐ human resources/payroll record
☐ other
☐ not stated

j) If other, please state

____

Please provide verbatim details of methods for VALUING other stated resource costs reported in paper

j) Actual stated costs of other resource/cost

____

Did the authors identify any cost drivers ?

◯ Yes ◯ No

If yes, please state the cost drivers reported in the study

Notes (please record any additional features of the study that deserve consideration when evaluating its quality)

____

Confirm paper not to be included in SR ◯ Yes ◯ No
