# Supplementary material for: The use of micro-costing in economic analyses of surgical interventions: a systematic review
Source: Health Econ Rev. 2020 Jan 29;10:3. doi: 10.1186/s13561-020-0260-8 (PMC6990532; doi:10.1186/s13561-020-0260-8)
Supplement: Supplementary file 4 — Additional file 4 Papers included in the systematic review (n = 85). [file 13561_2020_260_MOESM4_ESM.docx]

**Additional file 4: Papers included in the systematic review (n=85)**

1. Abbott MM, Alkire BC, Meara JG. The value proposition: using a cost improvement map to improve value for patients with nonsyndromic, isolated cleft palate. Plast Reconstr Surg. 2011;127(4):1650-1658.

2. Abbott MM, Meara JG. A microcosting approach for isolated, unilateral cleft lip care in the first year of life. Plast Reconstr Surg. 2011;127(1):333-339.

3. Abdelgadir J, Tran T, Muhindo A, et al. Estimating the Cost of Neurosurgical Procedures in a Low-Income Setting: An Observational Economic Analysis. World Neurosurg. 2017;101:651-657.

4. Aguilar-Bernier M, Gonzalez-Carrascosa M, Padilla-Espana L, Rivas-Ruiz F, Jimenez-Puente A, de Troya-Martin M. Five-year economic evaluation of non-melanoma skin cancer surgery at the Costa del Sol Hospital (2006-2010). J Eur Acad Dermatol Venereol. 2014;28(3):320-326.

5. Akhavan S, Ward L, Bozic KJ. Time-driven Activity-based Costing More Accurately Reflects Costs in Arthroplasty Surgery. Clin Orthop. 2016;474(1):8-15.

6. Alewijnse JV, van Rooijen EM, Kreulen M, Smeulders MJ, Tan SS. A microcosting study of the surgical correction of upper extremity deformity in children with spastic cerebral palsy. Dev Neurorehabil. 2017;20(3):173-178.

7. Ashraf A, Larson AN, Maradit-Kremers H, Kremers WK, Lewallen DG. Hospital costs of total hip arthroplasty for developmental dysplasia of the hip. Clin Orthop. 2014;472(7):2237-2244.

8. Au J, Rudmik L. Cost of outpatient endoscopic sinus surgery from the perspective of the Canadian government: a time-driven activity-based costing approach. Int Forum Allergy Rhinol. 2013;3(9):748-754.

9. Baratti D, Scivales A, Balestra MR, et al. Cost analysis of the combined procedure of cytoreductive surgery and hyperthermic intraperitoneal chemotherapy (HIPEC). Eur J Surg Oncol. 2010;36(5):463-469.

10. Berto P, Lopatriello S, Aiello A, et al. Cost of laparoscopy and laparotomy in the surgical treatment of colorectal cancer. Surg Endosc. 2012;26(5):1444-1453.

11. Chatterjee S, Laxminarayan R. Costs of surgical procedures in Indian hospitals. BMJ Open. 2013;3(6):20.

12. Chen A, Sabharwal S, Akhtar K, Makaram N, Gupte CM. Time-driven activity based costing of total knee replacement surgery at a London teaching hospital. Knee. 2015;22(6):640-645.

13. Coyan G, Wei LM, Althouse A, et al. Robotic mitral valve operations by experienced surgeons are cost-neutral and durable at 1 year. J Thorac Cardiovasc Surg. 2018;12:12.

14. Crott R, Lawson G, Nollevaux MC, Castiaux A, Krug B. Comprehensive cost analysis of sentinel node biopsy in solid head and neck tumors using a time-driven activity-based costing approach. Eur Arch Otorhinolaryngol. 2016;273(9):2621-2628.

15. Dassonville O, Bozec A, Chateau Y, et al. Multicenter prospective micro-costing study evaluating mandibular free-flap reconstruction. Eur Arch Otorhinolaryngol. 2017;274(2):1103-1111.

16. Dayananda K, Kong VY, Bruce JL, Oosthuizen GV, Laing GL, Clarke DL. Selective non-operative management of abdominal stab wounds is a safe and cost effective strategy: A South African experience. Ann R Coll Surg Engl. 2017;99(6):490-496.

17. de Paiva Haddad LB, Ducatti L, Mendes L, Andraus W, D'Albuquerque LAC. Predictors of micro-costing components in liver transplantation. Clinics. 2017;72(6):333-342.

18. Dionigi G, Bacuzzi A, Boni L, Rausei S, Rovera F, Dionigi R. Visualization versus neuromonitoring of recurrent laryngeal nerves during thyroidectomy: what about the costs? World J Surg. 2012;36(4):748-754.

19. Dombree M, Crott R, Lawson G, Janne P, Castiaux A, Krug B. Cost comparison of open approach, transoral laser microsurgery and transoral robotic surgery for partial and total laryngectomies. Eur Arch Otorhinolaryngol. 2014;271(10):2825-2834.

20. Essers BA, Dirksen CD, Nieman FH, et al. Cost-effectiveness of Mohs micrographic surgery vs surgical excision for basal cell carcinoma of the face. Archives of Dermatology. 2006;142(2):187-194.

21. Fanourgiakis J, Simantirakis E, Maniadakis N, et al. Cost-of-illness study of patients subjected to cardiac rhythm management devices implantation: results from a single tertiary centre. Europace. 2013;15(3):366-375.

22. Fawsitt CG, Bourke J, Greene RA, Everard CM, Murphy A, Lutomski JE. At what price? A cost-effectiveness analysis comparing trial of labour after previous caesarean versus elective repeat caesarean delivery. PLoS ONE. 2013;8(3):e58577.

23. Filetti S, Ladenson PW, Biffoni M, D'Ambrosio MG, Giacomelli L, Lopatriello S. The true cost of thyroid surgery determined by a micro-costing approach. Endocrine. 2017;55(2):519-529.

24. Traverso LW, Hargrave K. A prospective cost analysis of laparoscopic cholecystectomy. Am J Surg. 1995;169(5):503-506.

25. Tibesku CO, Hofer P, Portegies W, Ruys CJ, Fennema P. Benefits of using customized instrumentation in total knee arthroplasty: results from an activity-based costing model. Arch Orthop Trauma Surg. 2013;133(3):405-411.

26. Franchini M, Lippi G, Calzolari S, et al. Hysteroscopic Endometrial Polypectomy: Clinical and Economic Data in Decision Making. J Minim Invasive Gynecol. 2018;25(3):418-425.

27. Griffiths UK, Bozzani F, Muleya L, Mumba M. Costs of eye care services: prospective study from a faith-based hospital in Zambia. Ophthalmic Epidemiol. 2015;22(1):43-51.

28. Hamid KS, Matson AP, Nwachukwu BU, Scott DJ, Mather RC, 3rd, DeOrio JK. Determining the Cost-Savings Threshold and Alignment Accuracy of Patient-Specific Instrumentation in Total Ankle Replacements. Foot Ankle Int. 2017;38(1):49-57.

29. Head LK, McKay DR. Economic Comparison of Hand-Sutured and Coupler-Assisted Microvascular Anastomoses. J Reconstr Microsurg. 2018;34(1):71-76.

30. Heikkinen TJ, Haukipuro K, Koivukangas P, Hulkko A. A prospective randomized outcome and cost comparison of totally extraperitoneal endoscopic hernioplasty versus Lichtenstein hernia operation among employed patients. Surgical Laparoscopy and Endoscopy. 1998;8(5):338-344.

31. Heinz TR, Cowper PA, Levin LS. Microsurgery costs and outcome. Plast Reconstr Surg. 1999;104(1):89-96.

32. Herling SF, Palle C, Moller AM, Thomsen T, Sorensen J. Cost-analysis of robotic-assisted laparoscopic hysterectomy versus total abdominal hysterectomy for women with endometrial cancer and atypical complex hyperplasia. Acta Obstet Gynecol Scand. 2016;95(3):299-308.

33. Higgins KM. What treatment for early-stage glottic carcinoma among adult patients: CO2 endolaryngeal laser excision versus standard fractionated external beam radiation is superior in terms of cost utility? Laryngoscope. 2011;121(1):116-134.

34. Hii BW, McNab AA, Friebel JD. A comparison of external and endonasal dacryocystorhinostomy in regard to patient satisfaction and cost. Orbit. 2012;31(2):67-76.

35. Holschneider CH, Ghosh K, Montz FJ. See-and-treat in the management of high-grade squamous intraepithelial lesions of the cervix: a resource utilization analysis. Obstetrics and Gynecology. 1999;94(3):377-385.

36. Husby KR, Tolstrup CK, Lose G, Klarskov N. Manchester-Fothergill procedure versus vaginal hysterectomy with uterosacral ligament suspension: an activity-based costing analysis. Int Urogynecol J Pelvic Floor Dysfunct. 2018;26:26.

37. Ismail I, Wolff S, Gronfier A, Mutter D, Swanstrom LL. A cost evaluation methodology for surgical technologies.[Erratum appears in Surg Endosc. 2015 Aug;29(8):2433 Note: Swantrom, Lee L [corrected to Swanstrom, Lee L]; PMID: 25575907]. Surg Endosc. 2015;29(8):2423-2432.

38. Judd JP, Siddiqui NY, Barnett JC, Visco AG, Havrilesky LJ, Wu JM. Cost-minimization analysis of robotic-assisted, laparoscopic, and abdominal sacrocolpopexy. J Minim Invasive Gynecol. 2010;17(4):493-499.

39. Kanters TA, Wolff C, Boyson D, et al. Cost comparison of two implantable cardiac monitors in two different settings: Reveal XT in a catheterization laboratory vs. Reveal LINQ in a procedure room. Europace. 2016;18(6):919-924.

40. Kaur MN, Xie F, Shiwcharan A, et al. Robotic Versus Video-Assisted Thoracoscopic Lung Resection During Early Program Development. Ann Thorac Surg. 2018;105(4):1050-1057.

41. Khosravi MF, Janati A, Imani A, Javadzadeh A, Gharamaleki MM. Cost analysis of strabismus surgery by activity based costing. IIOAB Journal. 2016;7(10):63-69.

42. Koehler DM, Balakrishnan R, Lawler EA, Shah AS. Endoscopic Versus Open Carpal Tunnel Release: A Detailed Analysis Using Time-Driven Activity-Based Costing at an Academic Medical Center. J Hand Surg [Am]. 2018;2018:11.

43. Kolios L, Kolios G, Beyersdorff M, et al. Cost analysis of Topical Negative Pressure (TNP) Therapy for traumatic acquired wounds. Ger. 2010;8:Doc13.

44. Kong V, Aldous C, Handley J, Clarke D. The cost effectiveness of early management of acute appendicitis underlies the importance of curative surgical services to a primary healthcare programme. Ann R Coll Surg Engl. 2013;95(4):280-284.

45. Kramer EJ, Shearer DW, Marseille E, et al. The Cost of Intramedullary Nailing for Femoral Shaft Fractures in Dar es Salaam, Tanzania. World J Surg. 2016;40(9):2098-2108.

46. Krielen P, van den Beukel BA, Stommel MWJ, van Goor H, Strik C, Ten Broek RPG. In-hospital costs of an admission for adhesive small bowel obstruction. World J Emerg Surg. 2016;11:49.

47. Kupfer P, Abbott MM, Abramowicz S, Meara JG, Padwa BL. Cost differences between the anterior and posterior approaches to the iliac crest for alveolar bone grafting in patients with cleft lip/palate. J Oral Maxillofac Surg. 2012;70(3):685-689.

48. Lattimer CR, Azzam M, Kalodiki E, Shawish E, Trueman P, Geroulakos G. Cost and effectiveness of laser with phlebectomies compared with foam sclerotherapy in superficial venous insufficiency. Early results of a randomised controlled trial. Eur J Vasc Endovasc Surg. 2012;43(5):594-600.

49. Laviana AA, Ilg AM, Veruttipong D, et al. Utilizing time-driven activity-based costing to understand the short- and long-term costs of treating localized, low-risk prostate cancer. Cancer. 2016;122(3):447-455.

50. Lesurtel M, Selzner M, Petrowsky H, McCormack L, Clavien PA. How should transection of the liver be performed? A prospective randomized study in 100 consecutive patients: comparing four different transection strategies. Ann Surg. 2005;242(6):814-823.

51. Lince-Deroche N, Fetters T, Sinanovic E, Devjee J, Moodley J, Blanchard K. The costs and cost effectiveness of providing first-trimester, medical and surgical safe abortion services in KwaZulu-Natal Province, South Africa. PLoS ONE. 2017;12(4):e0174615.

52. Tan SS, van Putten E, Nijdam WM, et al. A microcosting study of microsurgery, LINAC radiosurgery, and gamma knife radiosurgery in meningioma patients. J Neurooncol. 2011;101(2):237-245.

53. Tan SS, Rutten FF, van Ineveld BM, Redekop WK, Hakkaart-van Roijen L. Comparing methodologies for the cost estimation of hospital services. Eur J Health Econ. 2009;10(1):39-45.

54. Taguchi K, Usawachintachit M, Tzou DT, et al. Micro-Costing Analysis Demonstrates Comparable Costs for LithoVue Compared to Reusable Flexible Fiberoptic Ureteroscopes. J Endourol. 2018;32(4):267-273.

55. Marino P, Houvenaeghel G, Narducci F, et al. Cost-Effectiveness of Conventional vs Robotic-Assisted Laparoscopy in Gynecologic Oncologic Indications. Int J Gynecol Cancer. 2015;25(6):1102-1108.

56. Marseille E, Kahn JG, Beatty S, Jared M, Perchal P. Adult male circumcision in Nyanza, Kenya at scale: the cost and efficiency of alternative service delivery modes. BMC Health Serv Res. 2014;14:31.

57. Martin JA, Mayhew CR, Morris AJ, Bader AM, Tsai MH, Urman RD. Using Time-Driven Activity-Based Costing as a Key Component of the Value Platform: A Pilot Analysis of Colonoscopy, Aortic Valve Replacement and Carpal Tunnel Release Procedures. J Clin Med Res. 2018;10(4):314-320.

58. McCreary DL, White M, Vang S, Plowman B, Cunningham BP. Time-Driven Activity-Based Costing in Fracture Care: Is This a More Accurate Way to Prepare for Alternative Payment Models? J Orthop Trauma. 2018;32(7):344-348.

59. Meier JD, Duval M, Wilkes J, et al. Surgeon dependent variation in adenotonsillectomy costs in children. Otolaryngol Head Neck Surg. 2014;150(5):887-892.

60. Meier JD, Zhang Y, Greene TH, Curtis JL, Srivastava R. Variation in pediatric outpatient adenotonsillectomy costs in a multihospital network. Laryngoscope. 2015;125(5):1215-1220.

61. Melfa GI, Raspanti C, Attard M, et al. Comparison of minimally invasive parathyroidectomy under local anaesthesia and minimally invasive video-assisted parathyroidectomy for primary hyperparathyroidism: a cost analysis. G Chir. 2016;37(2):61-67.

62. Mercier G, Naro G. Costing hospital surgery services: the method matters. PLoS ONE. 2014;9(5):e97290.

63. Mishra V, Geiran O, Krohg-Sorensen K, Andresen S. Thoracic aortic aneurysm repair. Direct hospital cost and Diagnosis Related Group reimbursement. Scand Cardiovasc J. 2008;42(1):77-84.

64. Mishra V, Tjonnfjord GE, Paus AC, Vaaler S. Orthopaedic surgery in severe bleeding disorders: a low-volume, high-cost procedure. Haemophilia. 2002;8(6):809-814.

65. Muralikrishnan R, Venkatesh R, Prajna NV, Frick KD. Economic cost of cataract surgery procedures in an established eye care centre in Southern India. Ophthalmic Epidemiol. 2004;11(5):369-380.

66. Soegaard R, Christensen FB, Christiansen T, Bunger C. Costs and effects in lumbar spinal fusion. A follow-up study in 136 consecutive patients with chronic low back pain. Eur Spine J. 2007;16(5):657-668.

67. Senna KM, Sarti FM, Costa MG, et al. Budget impact analysis of the percutaneous septal occluder for treatment of ostium secundum atrial septal defects in the Brazilian Unified National Health System. Cad Saude Publica. 2015;31(8):1756-1764.

68. Sebag F, Fortanier C, Ippolito G, Lagier A, Auquier P, Henry JF. Harmonic scalpel in multinodular goiter surgery: impact on surgery and cost analysis. J Laparoendosc Adv Surg Tech A. 2009;19(2):171-174.

69. Schreyogg J. A micro-costing approach to estimating hospital costs for appendectomy in a cross-European context. Health Econ. 2008;17(1 Suppl):S59-69.

70. Sabharwal S, Carter AW, Rashid A, Darzi A, Reilly P, Gupte CM. Cost analysis of the surgical treatment of fractures of the proximal humerus: an evaluation of the determinants of cost and comparison of the institutional cost of treatment with the national tariff. Bone Joint J. 2016;98-B(2):249-259.

71. Rossitto C, Gueli Alletti S, Romano F, et al. Use of robot-specific resources and operating room times: the case of Telelap Alf-X robotic hysterectomy. Int J Med Robot. 2016;12(4):613-619.

72. Ronkainen J, Blanco Sequeiros R, Tervonen O. Cost comparison of low-field (0.23 T) MRI-guided laser ablation and surgery in the treatment of osteoid osteoma. Eur Radiol. 2006;16(12):2858-2865.

73. Resnick CM, Daniels KM, Flath-Sporn SJ, Doyle M, Heald R, Padwa BL. Physician Assistants Improve Efficiency and Decrease Costs in Outpatient Oral and Maxillofacial Surgery. J Oral Maxillofac Surg. 2016;74(11):2128-2135.

74. Papakonstantinou VV, Kaitelidou D, Gkolfinopoulou KD, et al. Extracapsular hip fracture management: cost-consequences analysis of two alternative operative methods. Int J Technol Assess Health Care. 2008;24(2):221-227.

75. Paget JT, Young KC, Wilson SM. Accurately costing unilateral delayed DIEP flap breast reconstruction. Journal of Plastic, Reconstructive and Aesthetic Surgery. 2013;66(7):926-930.

76. Ottardi C, Damonti A, Porazzi E, et al. A comparative analysis of a disposable and a reusable pedicle screw instrument kit for lumbar arthrodesis: integrating HTA and MCDA. Health Econ Rev. 2017;7(1):17.

77. Nicod E, Jackson TL, Grimaccia F, et al. Direct cost of pars plana vitrectomy for the treatment of macular hole, epiretinal membrane and vitreomacular traction: a bottom-up approach. Eur J Health Econ. 2016;17(8):991-999.

78. Neyt MJ, Blondeel PN, Morrison CM, Albrecht JA. Comparing the cost of delayed and immediate autologous breast reconstruction in Belgium. Br J Plast Surg. 2005;58(4):493-497.

79. Narvy SJ, Ahluwalia A, Vangsness CT, Jr. Analysis of Direct Costs of Outpatient Arthroscopic Rotator Cuff Repair. Am J Orthop. 2016;45(1):E7-E11.

80. Assmann G, Kasch R, Hofer A, et al. An economic analysis of aseptic revision hip arthroplasty: calculation of partial hospital costs in relation to reimbursement. Archives of orthopaedic and trauma surgery. 2014;134(3):413-420.

81. Assmann G, Kasch R, Maher CG, et al. Comparison of health care costs between aseptic and two stage septic hip revision. The Journal of arthroplasty. 2014;29(10):1925-1931.

82. Kasch R, Merk S, Assmann G, et al. Comparative Analysis of Direct Hospital Care Costs between Aseptic and Two-Stage Septic Knee Revision. PloS one. 2017;12(1):e0169558.

83. Kasch R, Assmann G, Merk S, et al. Economic analysis of two-stage septic revision after total hip arthroplasty: What are the relevant costs for the hospital's orthopedic department? BMC musculoskeletal disorders. 2016;17:112.

84. Kasch R, Merk S, Drescher W, et al. Marginal contribution of UKS- versus TKA in varus arthritis of the knee. Archives of orthopaedic and trauma surgery. 2012;132(8):1165-1172.

85. Garrido-Gomez J, Arrabal-Polo MA, Giron-Prieto MS, Cabello-Salas J, Torres-Barroso J, Parra-Ruiz J. Descriptive analysis of the economic costs of periprosthetic joint infection of the knee for the public health system of Andalusia. The Journal of arthroplasty. 2013;28(7):1057-1060.
